# Association Among Professional Identity, Burnout, and Mental Health in Medical Students: A Cross-Sectional Study

**DOI:** 10.1097/ACM.0000000000006175

**Published:** 2025-07-22

**Authors:** Matteo Monti, Valerie Carrard, Céline Bourquin, Alexandre Berney

**Affiliations:** **M. Monti** is senior medical lecturer and senior physician, Medical Education Unit, University of Lausanne, and Division of Internal Medicine, Lausanne University Hospital, Lausanne, Switzerland; ORCID: https://orcid.org/0000-0002-7902-0607.; **V. Carrard** is senior research associate, Psychiatric Liaison Service, Lausanne University Hospital and University of Lausanne, Lausanne, Switzerland; ORCID: https://orcid.org/0000-0001-7355-9567.; **C. Bourquin** is associate professor and head of research, Psychiatric Liaison Service, Lausanne University Hospital and University of Lausanne, Lausanne, Switzerland; ORCID: https://orcid.org/0000-0001-9584-2929.; **A. Berney** is associate professor and senior physician, Psychiatric Liaison Service, Lausanne University Hospital and University of Lausanne, Lausanne, Switzerland; ORCID: https://orcid.org/0000-0001-9535-1222.

## Abstract

**Purpose:**

Professional identity formation in medical students is a multifactorial phenomenon in which clinical and nonclinical experiences merge with individual values, beliefs, and obligations. Although a strong professional identity has been associated with career success, a mismatch between personal orientations and expectations of the profession can create anxiety and feelings of inadequacy. Evidence exists of a possible association between professional identity and mental health or burnout in medical students. Nevertheless, literature on this topic is scant, and high-quality studies are lacking. Thus, the current study examined whether medical students’ professional identity is associated with mental health and burnout.

**Method:**

This cross-sectional study used data from the ETMED-L (Etudiants de Médecine-Lausanne) project collected between November 1 and December 2, 2021. All medical students at University of Lausanne across all study years were asked to complete validated questionnaires measuring professional identity, mental health (depressive symptoms, suicidal ideation, anxiety, and stress), and burnout (emotional exhaustion, cynicism, and academic efficacy).

**Results:**

In total, 1,033 medical students were included in the study. Professional identity was significantly and inversely related to depressive symptoms (*r* = −0.30), suicidal ideation (*r* = −0.34), and anxiety (*r* = −0.30). Professional identity was also significantly and inversely related to burnout: emotional exhaustion (*r* = −0.33), cynicism (*r* = −0.51), and academic efficacy (*r* = 0.45 [reversed dimension of burnout]). No significant differences in professional identity scores were observed between curriculum years.

**Conclusions:**

Medical students displaying higher professional identity also reported significantly fewer mental health issues and less burnout. Even though the cross-sectional design precludes any causal affirmation, the results suggest that high levels of professional identity may protect medical students against mental health issues and burnout. This study further warrants a multidimensional approach of professional identity to better capture its potential changes over time in future longitudinal studies.

Professional identity has been described as the “attitudes, values, knowledge, beliefs and skills that are shared with others within a professional group.”^1^ Professional identity formation in medical students is a multifactorial phenomenon in which clinical and nonclinical experiences, expectations, and environmental factors merge with individual values, beliefs, and obligations.^2–7^ The development of a strong professional identity has the potential to increase confidence and motivation, signal trustworthiness and expertise, and strengthen professional relationships.^8^ In the specific context of health care, professional identity formation is especially important because it is a necessary foundation to professionalism and could thus impact the quality of patient care.^9^ Although the formation of a professional identity in medical students has been associated with career success, a mismatch between personal orientations and expectations toward the profession has been associated with anxiety, frustration, feelings of inadequacy, and impostorship.^9^

A large body of literature suggests that medical students display more depression,^10^ suicidal ideation,^10^ anxiety,^11^ and burnout^12^ than aged-matched peers and have to deal with high levels of stress, contributing to pooled prevalences of 28.0% for depression,^10,13^ 11.1% for suicidal ideation,^10^ 33.8% for anxiety,^11^ and 44.2% for burnout.^14–17^ It has also been suggested that the same elements of the learning and working environment that promote burnout could also hinder the development of a well-integrated professional identity in medical students.^18^

However, studies examining the association between medical students’ professional identity and mental health or burnout are scant.^9^ Two studies, one among first-year junior physicians and the other among final-year medical students, have shown a positive association between high levels of professional identity and good mental health as well as between a low level of professional identity and personal and work burnout.^19,20^ A national survey among Chinese general practitioners also demonstrated a positive association of professional identity with job satisfaction and an inverse association with work burnout.^21^ Although the inverse association between professional identity and professional self-concept with burnout has also been described in other professions,^22–24^ literature concerning medical students is lacking. Some authors have suggested that the actual societal and work environment trends give excessive emphasis on technical aspects of medicine and foster detachment from patients and self-care, leading to burnout and deterioration in mental health and impairing medical student’s ability to develop a well-integrated professional identity.^18,25,26^ Literature also shows a complex intertwining between mental health or burnout and well-being, self-esteem, sense of accomplishment, work satisfaction, stress, and resilience. The latter are all factors that we can assume also influence the development of professional identity.^16,27,28^

Nevertheless, the paucity of high-quality studies undertaken on this topic and the heterogeneity of the existing literature in terms of target population, design, and instruments used to measure professional identity, burnout, or mental health do not allow for comparisons or confirmation of the associations or causal relationships. The current study took the opportunity of a large-scale study on interpersonal skills and mental health performed among a cohort of medical students of the 6-year curriculum^29^ to expand the body of knowledge on this topic. Using validated scales, this study examined the following research question: Is medical students’ professional identity associated with their mental health (depressive symptoms, suicidal ideation, anxiety, and stress) and burnout (emotional exhaustion, cynicism, and academic efficacy)? Given the theoretically positive impact of a strong professional identity,^8,9^ we hypothesized that medical students with higher professional identity scores would have fewer mental health issues and less burnout.

Both identity misalignment and mental health issues or burnout can lead to dissatisfaction and reduced ability to deal with stressors effectively,^30^ prompting many to leave the profession prematurely—an outcome the health care system can scarcely afford considering the current global physician shortage. Understanding the association among medical students’ professional identity, mental health, and burnout is a crucial first step in exploring whether, as some authors have suggested,^8^ professional identity can protect against the intense demands of medical training or whether mental health issues and burnout may disrupt the development of this identity.

## Method

### Population and procedure

This cross-sectional study used data collected as part of the ETMED-L (Etudiants de Médecine-Lausanne) project.^29^ In Switzerland, medical studies last 6 years and are divided into 3 preclinical, or bachelor, years (B1, B2, B3) and 3 master years (M1, M2, M3). M1 entails 4 months of clinical clerkships, and M3 entails 10 months of elective clinical rotations. The ETMED-L project investigates students’ mental health and interpersonal competences in the University of Lausanne Medical School among all medical students from B1 to M3, except foreign students registered as part of an academic exchange. This study used data from the ETMED-L project, collected between November 1 and December 2, 2021, during an exam-free month to minimize temporary stressors. The 2,096 eligible students from all 6 curriculum years received the invitation to participate and the link to the online survey through their academic email, and 2 reminder emails were subsequently sent. The online survey took approximately 60 minutes to complete, and students received a reward of U.S. $50 if they fully completed the survey. Participating students gave their informed consent through the online survey, and the study protocol was approved by the Human Research Ethics Committee of the Canton de Vaud.

### Measures

This study used validated measures of professional identity, mental health, and burnout, along with sociodemographic information, all of which were part of the ETMED-L questionnaire (available in full on Zenodo^31^).

#### Macleod Clark Professional Identity Scale.

The Macleod Clark Professional Identity Scale (MCPIS) was developed to investigate the professional identity of first-year health care students (including medical and social care students).^1^ It contains 9 statements to which participants indicate their agreement on a scale of 1 (totally agree) to 5 (totally disagree) and is the most researched and psychometrically validated professional identity scale for health care students.^32^ Given that no validated French version of the scale exists yet, a French version was created for the current study using a back-translation procedure. Similar to the previously published validation of the scale,^1,33,34^ exploratory factor analysis (principal component factor without rotation) showed that both a 1- and a 3-dimension structure of the MCPIS explained a satisfactory percentage of the variance and yielded good internal consistency. A confirmatory factor analysis (maximum likelihood) further indicated that the 3-dimension structure presented an acceptable fit to the data and should be preferred over the 1-dimension structure. However, the 3-dimension structure revealed 1 dimension with only 2 items and another dimension that was heavily skewed and with a method artifact (composed of all reversed items). Consequently, as suggested by the creators of the MCPIS, the 1-dimension structure was preferred,^1^ and the total sum score of the 9 items was used.

#### Mental health.

We used the validated French translation of the Center for Epidemiological Studies–Depression scale^35^ to assess self-reported depressive symptoms. This version demonstrated robust internal consistency and adequate structural and construct validity.^36^ Suicidal ideation was measured with 2 questions of the validated French version^37^ of the Beck Depression Inventory,^38^ which demonstrated significant diagnostic accuracy for indicating suicide ideation and previous or recent suicide attempts in university students.^39^ Anxiety was assessed with the validated French version^40^ of the trait section of the State-Trait Anxiety Inventory.^41^ This questionnaire, which measures participants’ general anxiety levels, has demonstrated robust construct and concurrent validity^42^ and the ability to predict caregiver distress.^43^ Stress levels were measured using a single generic item: Globally, how would you evaluate your current stress level on a scale of 1 (none) to 10 (extreme)?

#### Burnout.

The 3 dimensions of burnout were measured using the French version^44^ of the Maslach Burnout Inventory–Student Survey^45^: emotional exhaustion (5 items), cynicism (4 items), and academic efficacy (6 items, reversed dimension). This version demonstrated strong internal consistency and satisfactory structural validity.^44^

#### Covariates.

The following covariates were considered: curriculum year (B1, B2, B3, M1, M2, or M3), gender identity (male, female, or nonbinary), native language, having a partner (yes or no), having a paid job (yes or no), satisfaction with health (very unsatisfied to very satisfied), and having consulted a psychotherapist in the past year (yes or no).

### Analyses

Means (SDs), percentages, independent-sample *t* tests, and one-way analyses of variance (with Tukey post hoc tests) were used to describe our sample in terms of gender, curriculum year, native language, having a partner, having a paid job, satisfaction with health, and having consulted a psychotherapist last year. Then, Pearson correlations were run to test how medical students’ professional identity relates to their mental health (depressive symptoms, suicidal ideation, anxiety, and stress) and burnout (emotional exhaustion, cynicism, and academic efficacy). Effect sizes are considered small with a Pearson *r* = 0.1, medium with *r* = 0.3, and large with *r* = 0.5.^46^ Missing rates varied from 0% to 0.67% (with anxiety, stress, and burnout presenting the highest missing rates). Thus, listwise deletion was applied in the current study because imputation is unlikely to provide meaningful additional information with low missing rates (i.e., approximately 5%).^47^ All analyses were run using Stata software, version 16 (StataCorp, College Station, Texas).

## Results

Among the 2,096 eligible students who received an invitation to participate, 1,059 completed the survey. We additionally excluded 25 students who gave a wrong answer to at least 1 of the 2 attention questions placed in the survey (e.g., “In order to check your attention, please answer ‘Slightly agree’ to this question.”) and 1 student for whom the MCPIS was completely missing. The final sample thus included 1,033 students, which represents 49.28% of the eligible population.

### Study participant characteristics

The characteristics of the study participants are given in Table 1. The median (range) age of the sample is 22 (16–50) years. Participants were predominantly female. Female students did not significantly differ from male students in terms of professional identity (statistical comparison for nonbinary students was not reliable because the statistics represent only 1.26% of our sample). Professional identity varied significantly according to the degree of satisfaction with one’s health, with less satisfied students reporting lower professional identity (*r*_1033_ = 0.156, *P* < .001). Students who consulted a psychotherapist or psychiatrist in the past 12 months also reported lower professional identity (*t*_1031_ = 5.32, *P* < .001). Students’ professional identity did not significantly differ according to their native language, partnership status, or paid job status.

**Table 1:** Characteristics of University of Lausanne Medical Students in a Study of the Association of Professional Identity, Mental Health, and Burnout, November 1 to December 2, 2021

Characteristic	No. (%) of students	Professional identity score, mean (SD)
**Gender**		
Male	300 (29.04)	36.10 (5.27)
Female	720 (69.70)	35.99 (5.18)
Nonbinary	13 (1.26)	32.08 (6.08)
**Native language**		
French	829 (80.25)	35.88 (5.26)
Italian	56 (5.42)	36.02 (5.28)
Portuguese	31 (3.00)	36.55 (5.30)
English	29 (2.81)	36.93 (5.52)
German	24 (2.32)	35.25 (4.67)
Spanish	11 (1.06)	35.18 (5.02)
Serbian	7 (0.68)	36.14 (6.31)
Arab	7 (0.68)	39.57 (3.51)
Romanian	5 (0.48)	39.60 (2.07)
Croatian	3 (0.29)	35.00 (6.93)
Polish	3 (0.29)	35.00 (1.00)
Dutch	2 (0.19)	32.00 (4.24)
Russian	2 (0.19)	40.50 (2.12)
Czech	2 (0.19)	30.00 (7.07)
Turkish	2 (0.19)	38.00 (1.41)
Chinese	1 (0.10)	38.00 (0.00)
Finnish	1 (0.10)	34.00 (0.00)
Greek	1 (0.10)	41.00 (0.00)
Other	17 (1.65)	37.00 (5.09)
**Having a partner**		
No	493 (47.73)	35.71 (5.23)
Yes	540 (52.27)	36.22 (5.22)
**Having a paid job**		
No	689 (66.70)	36.13 (5.00)
Yes	344 (33.30)	35.65 (5.65)
**Satisfaction with health**		
Very dissatisfied	23 (2.23)	36.52 (4.97)
Dissatisfied	103 (9.97)	33.65 (6.62)
Neither satisfied nor dissatisfied	180 (17.42)	35.22 (4.98)
Satisfied	503 (48.69)	36.21 (5.01)
Very satisfied	224 (21.68)	37.05 (4.83)
**Consulted a psychiatrist in the past year**		
No	752 (72.80)	36.49 (5.04)
Yes	281 (27.20)	34.58 (5.47)

### Association among professional identity, mental health, and burnout

Associations among medical students’ professional identity, mental health, and burnout are presented in Table 2. Professional identity was significantly and inversely related to mental health issues, with effect sizes that were low for stress (*r* = −0.15) and medium for depressive symptoms (*r* = −0.30), suicidal ideation (*r* = −0.34), and anxiety (*r* = −0.30; *P* < .001 for all). Professional identity was also inversely related to burnout, with effect sizes that were medium for emotional exhaustion (*r* = −0.33) and academic efficacy (*r* = 0.45 [reversed dimension]) and large for cynicism (*r* = −0.51; *P* < .001 for all).

**Table 2:** Descriptive and Pearson Correlations of University of Lausanne Medical Students’ Professional Identity With Their Mental Health and Burnout, November 1 to December 2, 2021

Outcome	No. of students	Mean (SD) score [range]	Skewness^a^	Kurtosis^a^	Cronbach *α*	Correlations (*r*) with professional identity^b^
**Professional identity**	1,033	35.97 (5.23) [14–45]	−0.92	4.05	0.78	NA
**Mental health**						
Depressive symptoms	1,028	19.53 (11.41) [0–58]	0.61	2.86	0.93	−0.30^c^
Suicidal ideation	1,028	0.73 (1.07) [0–5]	1.54	4.89	0.49	−0.34^c^
Anxiety	1,027	44.64 (12.12) [20–80]	0.11	2.36	0.94	−0.30^c^
Stress	1,027	5.56 (2.20) [1–10]	−0.26	1.96	NA	−0.15^c^
**Burnout**						
Emotional exhaustion	1,027	16.79 (4.94) [5–30]	0.13	2.73	0.86	−0.33^c^
Cynicism	1,027	9.59 (4.49) [4–24]	0.88	3.34	0.86	−0.51^c^
Academic efficacy	1,027	23.89 (4.62) [8–36]	−0.16	2.80	0.76	0.45^c^

Abbreviation: NA, not applicable.

^a^Absolute values of skewness larger than 2 and of kurtosis larger than 7 indicate substantial nonnormality.^46^

^b^Correlations of 0.1, 0.3, and 0.5 indicate small, medium, and large effect sizes, respectively.^44^

^c^*P* < .001.

### Professional identity and curriculum years

We found no differences regarding professional identity among curriculum years that could indicate a linear change from year to year. The only significant differences in terms of professional identity were that B3 students reported significantly higher professional identity scores compared with B1 students (*t*_1027_ = 2.93, *P* = .04) and M2 (*t*_1027_ = 3.34, *P* = .01) students (Table 2). Thus, we ran additional exploratory analyses separately for the 3 potential factors of professional identity. Indeed, as described in the Measures section, past psychometric testing of the MCPIS in samples of health and social work students^1,33^ as well as our own psychometric test of the scale indicate that the MCPIS might include 3 distinct dimensions of professional identity. On the basis of the items, we labeled these dimensions sense of membership, negative attitudes (reversed dimension), and positive attitudes (see Table 3 for the items of each dimension). These 3 dimensions did not yield similar results when comparing the scores of different curriculum years. Compared with students in earlier curriculum years, students in later curriculum years scored significantly higher in the sense of membership dimension (*F*_5,1027_ = 35.48, *P* < .001) but also scored higher in the dimension of negative attitudes (*F*_5,1027_ = 7.16, *P* < .001) and lower in the dimension of positive attitudes toward the medical profession (*F*_5,1027_ = 10.11, *P* < .001).

**Table 3:** ANOVAs With Tukey Post Hoc Tests Comparing University of Lausanne Medical Students’ Professional Identity Across Curriculum Years, November 1 to December 2, 2021

Outcome		ANOVAs			Contrasts (*t* test)				
	Year^a^	No. of students	Mean (SD)	*F*	vs B2	vs B3	vs M1	vs M2	vs M3
**Professional identity total score**	B1	302	35.57 (4.89)	3.20^b^	1.30	2.93^c^	1.81	−0.97	0.00
	B2	156	36.24 (4.68)			1.39	0.48	−1.95	−1.06
	B3	168	37.04 (4.67)				−0.87	−3.34^c^	−2.39
	M1	142	36.53 (4.55)					−2.38	−1.50
	M2	141	35.06 (5.84)						0.81
	M3	124	35.57 (6.89)						
**Sense of membership dimension (Cronbach *α* = 0.80)** 1. I feel like I am a member of this profession. 2. I feel I have strong ties with members of this profession.	B1	302	4.73 (2.26)	35.48^d^	4.23^d^	7.70^d^	7.75^d^	9.03^d^	11.09^d^
	B2	156	5.56 (1.85)			2.92^c^	3.20^c^	4.34^d^	6.37^d^
	B3	168	6.20 (1.82)				0.41	1.58	3.73^c^
	M1	142	6.30 (1.74)					1.12	3.21^b^
	M2	141	6.56 (1.88)						2.13
	M3	124	7.08 (2.03)						
**Negative attitudes dimension (Cronbach *α* = 0.73)** 3. I am often ashamed to admit that I am studying for this profession. 4. I find myself making excuses for belonging to this profession. 5. I try to hide that I am studying to be part of this profession.	B1	302	3.91 (1.78)	7.16^d^	0.18	0.72	0.63	4.73^d^	3.89^b^
	B2	156	3.95 (1.87)			0.47	0.40	4.00^d^	3.30^c^
	B3	168	4.05 (1.99)				−0.05	3.62^b^	2.92^c^
	M1	142	4.04 (1.70)					3.52^c^	2.85
	M2	141	4.89 (2.49)						−0.55
	M3	124	4.75 (2.46)						
**Positive attitudes dimension (Cronbach *α* = 0.79)** 6. I am pleased to belong to this profession. 7. I can identify positively with members of this profession. 8. Being a member of this profession is important to me. 9. I feel I share characteristics with other members of the profession.	B1	302	16.76 (2.63)	10.11^d^	−0.47	0.50	−1.70	−4.82^d^	−5.08^d^
	B2	156	16.63 (2.59)			0.85	−1.09	−3.83^b^	−4.11^b^
	B3	168	16.89 (2.36)				−1.94	−4.72^d^	−4.98^d^
	M1	142	16.27 (2.66)					−2.68	−3.00^c^
	M2	141	15.38 (3.14)						−0.41
	M3	124	15.24 (3.63)						

Abbreviation: ANOVA, analysis of variance.

^a^In Switzerland, medical studies last 6 years and are divided into 3 preclinical, or bachelor, years (B1, B2, B3) and 3 master years (M1, M2, M3).

^b^*P* < .01.

^c^*P* < .05.

^d^*P* < .001.

### Sensitivity analyses

Sensitivity analyses showed that the correlations between the professional identity total score and the indicators of mental health and burnout remained similar in terms of significance and effect size magnitude when controlling for students’ gender and curriculum year, except for anxiety, which became nonsignificant (partial correlations, see Supplemental Digital Appendix 1 at http://links.lww.com/ACADMED/B755). Likewise, when calculating the Spearman rank correlations to account for potential nonnormality issues, we observed similar significances and effect size magnitudes (see Supplemental Digital Appendix 2 at http://links.lww.com/ACADMED/B755).

Moreover, when analyzing the 3 dimensions of professional identity separately, we observed similar results in terms of significance and direction for the sense of membership and negative attitudes (Table 4). More sense of membership and fewer negative attitudes were indeed significantly and consistently related to fewer health issues and less burnout, although with smaller effect sizes than for the professional identity total score. For positive attitudes, results indicate that professional identity is the dimension most strongly negatively related to burnout (and especially cynicism), but it is not significantly related to anxiety or stress.

**Table 4:** Pearson Correlations of the Professional Identity’s Subdimensions With University of Lausanne Medical Students’ Mental Health and Burnout, November 1 to December 2, 2021

Outcome	No. of students	Sense of membership	Negative attitudes	Positive attitudes
**Mental health**				
Depressive symptoms	1,028	−0.31^a^	0.19^a^	−0.17^a^
Suicidal ideation	1,028	−0.25^a^	0.24^a^	−0.25^a^
Anxiety	1,027	−0.28^a^	0.15^b^	0.04
Stress	1,027	−0.18^a^	0.13^a^	−0.04
**Burnout**				
Emotional exhaustion	1,027	−0.27^a^	0.22^a^	−0.24^a^
Cynicism	1,027	−0.24^a^	0.35^a^	−0.50
Academic efficacy	1,027	−0.30^a^	−0.26^a^	0.41^a^

^a^*P* < .001.

^b^*P* < .05.

## Discussion

We observed consistent significant associations among professional identity, mental health, and burnout in a cohort of more than 1,000 medical students. Medical students with higher scores of professional identity reported significantly fewer mental health issues (depressive symptoms, suicidal ideation, anxiety, and stress), were more satisfied with their health, and were less likely to have consulted a psychotherapist or psychiatrist in the past 12 months. Given the cross-sectional nature of the current study, the causality of this association between professional identity and mental health remains uncertain. Previous observations indicate that individuals with a low sense of shared identity—and consequently lower group engagement—experience reduced supportive cooperation and are more susceptible to psychological distress,^48^ thus supporting that a lack of professional identity might trigger mental health issues. However, the reverse causation, that mental issues preclude the development of a strong professional identity, is equally supported by the literature reporting that psychological distress negatively impacts social interactions and the perception of support,^49^ thereby hindering the development of a solid professional identity. More studies, and especially longitudinal ones, are needed to clarify whether professional identity could be targeted to protect the mental health of medical students or if professional identity is rather a symptom of medical students’ mental health status.

An inverse correlation with professional identity was similarly found for burnout. This association was also observed in a study of first-year junior physicians in which participants with a stronger professional identity reported lower levels of cynicism and emotional exhaustion, along with a higher sense of personal accomplishment (or academic efficacy).^20^ The association between professional identity and cynicism was especially strong in our study and seems pertinent. Indeed, the development of a professional identity requires students to engage in a series of negotiations to reconcile their personal values with the norms and values of their profession and community of practice,^50^ whereas cynicism may reflect a struggle to put a proper distance between themselves, their idealized concepts of health care, and the complex and stressful learning and work environment.^51^ This finding suggests that emphasizing professional identity development within the curriculum may reduce cynicism among medical students. However, it might also be that cynicism itself prevents the development of a strong professional identity. Cynicism could indeed reflect the poor quality of social relationships in the working environment and the lack of critical capacities,^52^ which should prompt targeted strategies to monitor and support students. We also found an inverse correlation between emotional exhaustion and professional identity. It has been suggested that exhaustion in physicians and medical students originates from stressful societal and learning environments, which diminish their ability to recognize their own feelings and emotional needs and to alleviate distress. This reduced self-care may explain why medical students struggle to develop a solid professional identity.^18,25,26^ This result may highlight the importance of reinforcing self-care education and efforts to enhance learning and working environments as supportive elements of professional identity development. Regarding the association we found between professional identity and academic efficacy, it aligns with previous studies among health professionals and social workers, showing that a strong professional identity is associated with higher levels of accomplishment.^53–55^ In addition, social belonging,^56^ a component of professional identity, predicts motivation and achievement, protects against burnout,^57^ and reduces stress.^58^ Overall, further investigations are needed to disentangle the potential mediating factors that could explain the associations between professional identity and the 3 different dimensions of burnout. Potential candidates include vulnerability to stress that could hinder professional identity development and trigger burnout, resilience that could participate in the development of a strong professional identity and protect from burnout, work satisfaction that has been related to stronger professional identity and less burnout, and social support, which is a vector of stronger professional identity and a protective factor against burnout.^16,27^

The other important finding emerging from this study is that the level of professional identity, when measured with the MCPIS total score, did not differ among the curriculum years. Medical students scored highest at the professional identity scale as they entered their studies, suggesting a strong overall identification already at early stages of training.^1,59,60^ The lack of differences among curriculum years is surprising, considering that professional identity has been described as a dynamic evolving process.^61,62^ A previous 1-year longitudinal study among medical students using the same scale also found a stability of professional identity levels from before to after graduation.^20^ We found a possible explanation through a multidimensional analysis of the MCPIS scores. Indeed, considering the MCPIS as a 3-dimension construct (see the Measures section and Table 3), we found significant differences among the study years. Students in later years presented not only a significantly higher sense of membership to the profession but also a more negative and less positive attitude toward the profession than students in earlier years. These results question the 1-dimensionality of the MCPIS and/or the ability of the total score to accurately capture professional identity as a process in formation. However, our analyses are cross-sectional, and definite conclusions on the matter should await longitudinal findings.

### Study implications

The demonstration of an association among professional identity, mental health, and burnout among medical students should stimulate research to elucidate the causal processes underlying it. Our results should ultimately prompt program directors and medical educators to implement concrete actions to support the development of a well-integrated professional identity in medical students and the support of medical students’ psychological well-being to nurture future physicians who are better prepared to face the challenges of medical practice. Concrete actions include the valorization of positive role models and the provision of a supportive learning environment that can mitigate negative influences on core values, thereby reducing the risk of burnout.^63^

The current study’s results warrant a multidimensional approach to professional identity to better capture potential intervention effectiveness. The diverging results pertaining to the 3 subdimensions of professional identity in terms of curriculum year differences call for further explorations of the MCPIS as a multidimensional measure, which might be better suited to assess professional identity as a dynamic and complex process. However, the psychometric properties of the subdimensions would first need to be improved in terms of number of items per dimensions and method artifact (see Measures section).

### Limitations and strengths

The lack of a validated French version of the MCPIS is regrettable. However, the back-translation procedure applied in the current study was thorough, and the fact that our own exploration of the scale’s factor structure replicated the one published by its creators supports the accuracy of our translation. The monocenter design of the study could limit the generalizability of its results. This limitation could be mitigated in part by the large sample of students from a 6-year curriculum cohort and the use of validated scales, but additional multisite studies are warranted to ascertain generalizability and understand the potential impact of specific educational environments. The U.S. $50 reward given to the participating students comes with upsides and downsides. It may explain the high study response rate and have increased the participation of individuals with poorer health and more socioeconomic difficulties who are known to participate less to cohort studies.^64^ However, financial retribution comes with the risk of lack of attention of students participating solely for monetary purposes. The attention questions placed in the survey were designed to mitigate this effect. The cross-sectional design did not allow for the testing of causal relationships or the estimation of the longitudinal trajectories of the different dimensions of professional identity, both aspects that were beyond the scope of this study. However, results open interesting directions for future longitudinal studies to determine the causal relationship among professional identity dimensions, mental health, and burnout among medical students. Additional data collections within the ETMED-L project are planned, which we hope will shed light on this question.

### Conclusion

Using a range of validated questionnaires, we found significant associations among professional identity, mental health, and burnout among medical students. A high level of professional identity may be protective of students’ mental health and burnout, whereas a low level is associated with more anxiety, depression, suicidality, stress, and burnout. Given the potential consequences of a poorly developed professional identity, program directors, clinical supervisors, and deaneries should intensify efforts to monitor the learning environment, valorize positive role models, and develop activities aimed at facilitating harmonious professional identity development. The extent to which professional identity evolves during medical studies and the analysis of factors that influence the association between professional identity and mental health needs to be explored in future longitudinal studies. Understanding the association among professional identity, mental health, and burnout, and ultimately their interinfluences, would enable organizations to design curricula and services that build on physicians’ strengths, promoting identity coherence and providing resources to support well-being.
